# Frontier Progress of Unmanned Aerial Vehicles Optical Wireless Technologies

**DOI:** 10.3390/s20195476

**Published:** 2020-09-24

**Authors:** Jupeng Ding, Hongye Mei, Chih-Lin I, Hui Zhang, Wenwen Liu

**Affiliations:** 1 Key Laboratory of Signal Detection and Processing in Xinjiang Uygur Autonomous Region, School of Information Science and Engineering, Xinjiang University, Urumqi 830046, Xinjiang, China; meihongye@stu.xju.edu.cn (H.M.); wwliu@stu.xju.edu.cn (W.L.); 2 China Mobile Research Institute, Beijing 100053, China; icl@chinamobile.com; 3 Xinjiang Vocational & Technical College of Communications, Urumqi 831401, Xinjiang, China; zh007red@163.com

**Keywords:** optical wireless communications, unmanned aerial vehicle, free space optics, visible light communications, optical communications link

## Abstract

With the continuous maturity of unmanned aerial vehicles (UAV) in materials, communications, and other related technologies, the UAV industry has developed rapidly in recent years. In order to cope with the diversified emerging business forms, the explosive growth of the scale of data traffic, number of terminal connections, high reliability, low-latency, and high transmission rate provided by the fifth generation (5G) network will inject new vitality into the development of the UAVs industry. In this paper, optical wireless technology is introduced into the UAV platform, combining theory with practical applications. We explain many research advances and key technologies in the four aspects of “air, space, earth, and sea” to achieve a strong and broadband communication link. This discussion focuses on link modeling, parameter optimization, experimental testing, and the status quo of UAVs in different application scenarios with optical wireless link configurations. At the same time, based on the current situation of UAV optical wireless technology, the technical problems and the research direction in the future are also discussed.

## 1. Introduction

**Figure 1:**
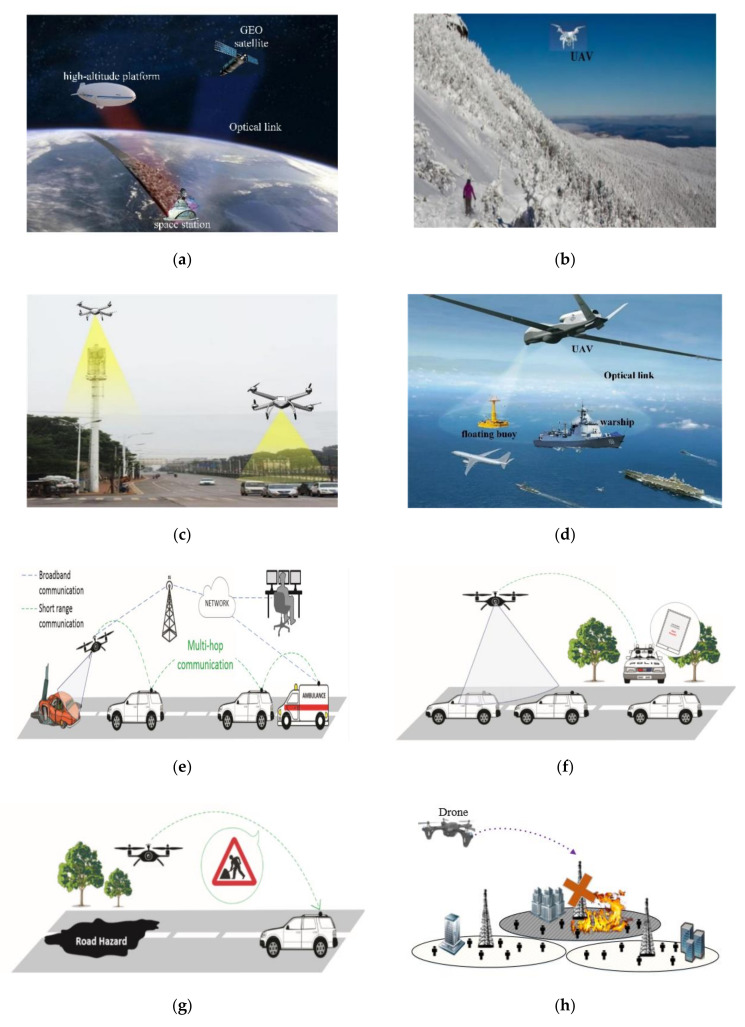
Unmanned aerial vehicle (UAV) application scenarios. (**a**) UAV–satellite communication; (**b**) disaster relief scene; (**c**) UAV–ground communication; (**d**) UAV–sea surface communication; (**e**) a UAV is used to provide the rescue team with an advance report prior to reaching the incident scene; (**f**) a UAV is used by police to catch traffic violations; (**g**) a UAV is used as a flying roadside unit (RSU) that broadcasts a warning about road hazards that have been detected in an area not pre-equipped; and (**h**) a scene of a fire [3,4].

With the advent of fifth generation (5G), this technology is expected to significantly increase capacity and new applications for large-scale connections. The 5G wireless system is still ground-based and has the same coverage complexity as other terrestrial networks. However, in the face of the destruction of ground infrastructure caused by sudden disasters, these ground coverage networks will be damaged to varying degrees or even become unavailable. The space communication network is a supplement to the terrestrial communication network. It can not only provide extensive communication coverage for people and vehicles in the sea, remote rural areas, and the air, but also provide timely network connections when the ground network is damaged. Figure 1 shows some typical communication network application scenarios. Compared with radio frequency (RF) communication technology widely used in 5G, optical wireless communication (OWC) technology is not restricted by frequency band, has fast transmission rate and low cost, and can become the key technology of the sixth generation (6G) communication system. Secondly, unmanned aerial vehicles (UAVs) have been greatly improved in technology and cost, and are gradually becoming a mobile communication terminal to access the communication network. Obviously, the future information network must realize the seamless integration of the space network and ground network. The envisaged 6G wireless system is expected to support true global wireless communication anytime and anywhere [1,2]. How to build a high-capacity but low-cost communication system is of great significance to the development of global 6G communication systems and OWC technology based on UAVs may become one of the important solutions for 6G wireless systems.

### 1.1. Unmanned Aerial Vehicle

UAV technology has extended from the traditional military field to many civilian fields such as agriculture, security, surveillance, and commodity delivery. Considering that UAVs often need to interact with high speed data, traditional RF communication links can no longer independently support the above applications. Especially in special situations such as emergency rescue, UAVs can use traditional wireless links or satellite communications to cooperate with ground terminals (including manned and unmanned vehicles, crawling robots, handheld devices, etc.) before they can obtain information from UAVs. The global vision is transformed into the analysis and judgment of ground targets, and the ground terminal is dynamically guided over obstacles for accurate rescue and repair. It must be pointed out that traditional links are subject to factors such as capacity, interference, confidentiality, and spectrum resources; satellite communications are subject to factors such as high cost, interference, and time delay, and cannot fully meet the communication needs of air-to-ground (A2G) UAV heterogeneous systems [5,6,7,8,9].

**Figure 2:**
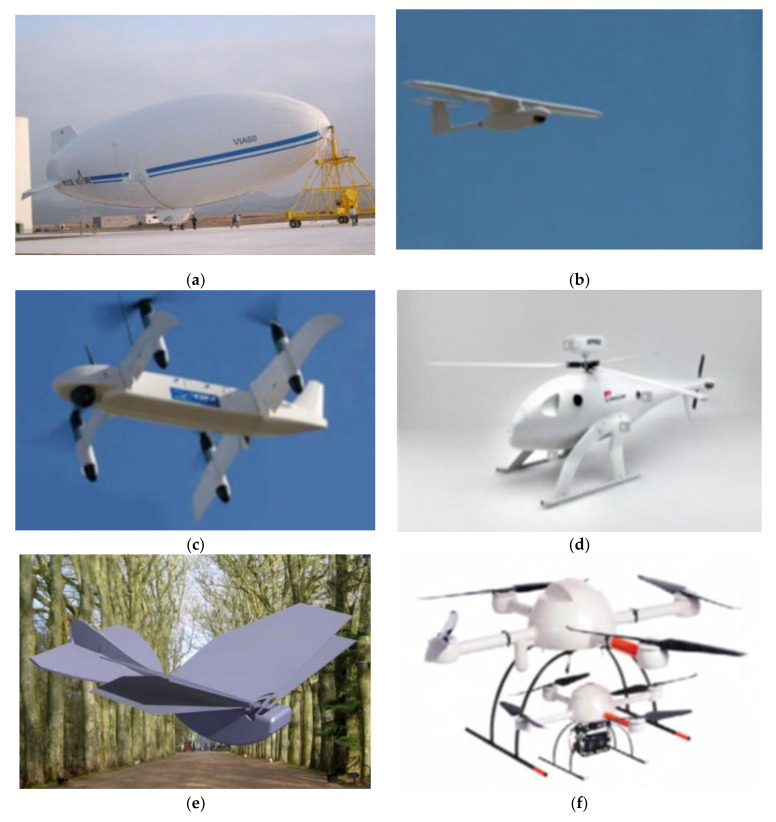
Main types of UAVs: (**a**) unmanned airship; (**b**) fixed-wing; (**c**) tilt-wing; (**d**) unmanned helicopter; (**e**) flapping-wing; and (**f**) multi-copter [19,20].

At the same time, the range of UAVs available is being further expanded, and can be divided into high-altitude platform (HAP), fixed-wing, inclined wing, helicopter type, ornithopter type, and multi-rotor type, as shown in Figure 2. An HAP is a quasi-static aircraft similar to an airship, usually filled with helium and continuously operating in the stratosphere. The fixed-wing UAV has excellent endurance and flight range, but it must take off horizontally, which requires more space for free flight and is more suitable for high altitude applications. Because of its hybrid wing design, tilt-wing UAVs support vertical take-off and landing while continuing the traditional advantages of fixed-wing UAVs; it is important to note that, high cost and technical complexity are the main constraints to its widespread commercialization. On the one hand, helicopter UAVs can support vertical take-off and landing, as well as high operability and payload capacity; similarly, the relatively high cost of construction and maintenance also prevents them from becoming the preferred platform for UAVs. Flapping-wing, also named ornithopter, can integrate a power system and control system without vertical take-off and landing, and its mechanical efficiency is high. However, the ornithopter aerodynamics are not mature enough to guide the design of aircraft, which makes the ornithopter difficult to be used for high speed and large scale applications. On the other hand, multi-rotor UAV is the first choice for most low-altitude platform (LAP) UAV applications due to its excellent performance at low cost, easy takeoff and landing, and low weight. At present, researchers for OWC technology in the field of UAVs are more focused on this type of aircraft.

### 1.2. Optical Wireless Communication Technology

OWC technology is a general term for a kind of optical communication technology that uses free space as a transmission medium. Since most OWC technologies do not require extensive infrastructure, installation costs can be minimized [10]. Since light cannot penetrate the surrounding walls, OWC can support improved data security. In addition, this type of technology also has many advantages such as high propagation direction, large transmission capacity, strong confidentiality, no need for spectrum authorization, and outstanding anti-interference ability [11,12]. In OWC, visible light, infrared radiation, or ultraviolet spectrum is used as the propagation medium. Many wireless systems are being developed based on these three optical bands. At present, the most promising optical communication technologies include visible light communication (VLC), light fidelity (LiFi), optical camera communication (OCC), and free space optical (FSO) communication [13]. The communication media, communication protocol, architecture, and application scenarios of each technology are different. On the one hand, in indoor scenarios, light-emitting diode (LED) lights are gradually replacing laser sources. Loading data for the LED light source through the drive circuit can not only achieve indoor lighting but also provides data coverage [14,15]. So far, VLC has received extensive attention from manufacturers, and academia has established research institutions and research organizations. On the other hand, OWC technology has also made a certain degree of progress in outdoor scenarios, and it has played a unique role that cannot be replaced in many application fields such as wireless backhaul, satellite interconnection, emergency support, and temporary links [16,17,18].

Traditional optical communication uses intensity modulation (IM) and Mach–Zehnder modulation (MZM), IM only uses amplitude modulation. This modulation format is easy to implement, requires relatively simple equipment, and is inexpensive. However, in recent years, with the high-capacity long-distance optical wireless transmission system, a variety of new light modulation formats have been proposed and applied. This includes on/off keying (OOK) control based on intensity modulation, phase shift keying (PSK) based on phase modulation, and quadrature amplitude modulation (QAM) based on both control amplitude and phase. Table 1 is a simple summary of modulation methods in recent years [21,22,23,24,25,26,27,28,29,30,31]. Optical signals are unipolar in comparison to RF systems, and any bipolar scheme requires the light source to be biased with the help of a direct current (DC) bias. Therefore, compared with OOK, the QAM scheme with more efficient bandwidth has bias penalty, which weakens the advantages of the QAM scheme to some extent. However, in the VLC system, bi-polar orthogonal frequency division multiplexing (OFDM) signals cannot be loaded directly onto the LED. In order to ensure the non-negative nature of transmission signal, researchers propose VLC asymmetrically clipped optical orthogonal frequency division multiplexing (ACO-OFDM) and direct current orthogonal frequency division multiplexing modulation (DCO-OFDM) technology [23,32,33,34,35,36].

The integration of OWC technology and UAV flight platforms is mainly oriented to UAV–satellite terminals, UAVs, UAV–ground terminals, and UAV–sea surface terminals. At present, the requirement of a communication link between UAV and the ground terminal is the most studied, which is often called the A2G problem. On the one hand, the fusion solution for this demand can simultaneously leverage the comprehensive advantages of UAVs, such as high spatial freedom, short deployment time, strong optical wireless technology positioning, and large capacity. Through the construction of airmobile relay, the fast establishment of a high capacity transmission link between fixed ground terminals is realized. On the other hand, in the application fields such as disaster relief, security, and transportation, UAVs often need to complete high-speed data transmission and instruction issuing with mobile terminals such as vehicles and robots [7,29,37,38,39,40,41,42,43]. For this kind of scenario, the mobile characteristics of UAVs and ground terminals must be fully considered, and a solution with high applicability and high availability is proposed to truly release the potential and efficiency of this kind of fusion technology. Based on the above considerations, the industry proposes to introduce OWC technology into the field of UAVs, to greatly improve the wireless transmission capacity of UAVs by mining rich spectral resources. Based on the original UAV–satellite link, UAVs link, UAV–ground terminal link, and UAV–sea surface link, optical wireless modules and subsystems are integrated into various UAVs and terminals respectively, to build a light-wave-based link with high speed, high security, and orientation [25,38,39,40,41,42].

### 1.3. Contributing to This Article

This paper briefly describes the current situation and forward progress of OWC technology based on the UAV platform from four perspectives: UAV–satellite terminal, UAVs, UAV–ground terminal, UAV–sea surface terminal. This paper analyzes many technical challenges faced by UAVs to achieve reliable, high-speed optical wireless links. At the same time, it summarizes a series of targeted research works that have been carried out in academia and industry.

## 2. Unmanned Aerial Vehicle (UAV)-Satellite Optical Wireless Communications

The coverage of terrestrial 5G networks is usually limited, far from being able to meet the communication needs of remote rural areas, post disaster, and maritime communications. In the 6G era, both satellites and drones can be used to solve this problem. At present, researchers at home and abroad are actively exploring the construction of broadband OWC between UAVs and satellites. The special form of unmanned system has also received extensive attention and discussion [44,45]. Correspondingly, how to establish a highly reliable optical wireless communication link between the high-altitude platform and the artificial satellite (also named air-to-satellite (A2S) links), how to effectively control or even overcome the adverse effects of absorption, scattering, beam broadening, phase front distortion, and various weather phenomena on the link performance, and how to build an optical wireless network integrating air, space, earth, and sea is gradually becoming the focus of research.

### 2.1. Link Modeling and Characteristic Analysis

**Figure 3:**
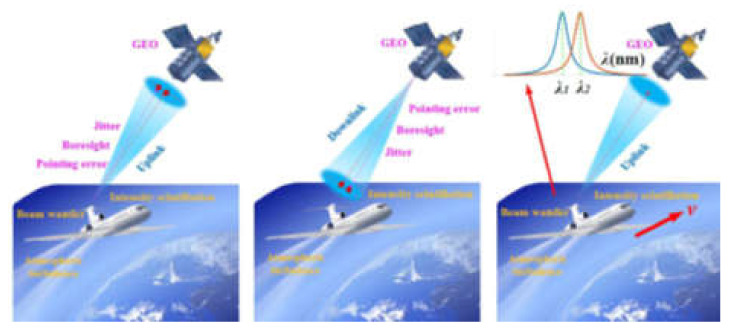
Illustration of the optical uplink, optical downlink, and the Doppler effects between UAV.

In recent years, extensive research has been carried out to improve the quality of communication services and the deployment of emergency networks in case of emergencies. In the event of a catastrophic event, HAPs can be equipped with telecommunications equipment to support these critical communications tasks; having HAPs in the area to observe and sense the need to take a lot of high-definition pictures. Considering the storage and long transmission of UAVs, Mi Li et al. from Nanjing University proposed to construct an optical wireless link between UAVs and satellites. UAVs are used as communication relays to first send data to satellites and then to ground stations, to improve the overall performance of data return [46]. The communication link is mainly affected by the Doppler effect, pointing error, and atmospheric turbulence effect. In the case that HAP alone is considered, Kandeepan discusses a real-time adaptive collaborative transmission strategy that dynamically selects direct and collaborative links based on channel conditions to improve transmission efficiency. He emphasized that such a cooperative link can improve the energy efficiency of the uplink adaptive cooperative scheme, which is mainly used to extend the survivability of the battery in the air–ground communication link [41]. In addition, different from the high stability of ground station terminals, UAVs as communication terminals will inevitably introduce pointing errors of different degrees. When a UAV is used as a communication terminal, the UAV and artificial satellites cannot stay relatively still, so researchers must fully consider the Doppler effect introduced by the relative motion between the two. Specifically, Figure 3 shows the schematic diagram of optical downlink, uplink, and Doppler effect between the UAV and artificial satellites [46]. In terms of uplink, Mi Li et al. obtained the joint probability distribution of beam center deviation caused by pointing error and beam drift effect by convolution. Regarding the aspect of downlink, this paper presents a mathematical model which is similar to the traditional air–ground link. According to the relevant literature, Table 2 summarizes the progress made in the optical communication link between UAVs and satellites in recent years. We can see that under the optical wireless link condition between a UAV and a satellite at a height of 36,000 km, V. Cazaubiel realized the uplink of 2 Mb/s and the downlink of 50 Mb/s [33]. In the experimental environment [47,48,49,50,51], the transmission rate in the magnitude of Gb/s has been realized, and the above experiments have preliminarily confirmed the availability of an optical wireless link between UAV and satellite.

### 2.2. High Altitude Platform Unmanned System

**Figure 4:**
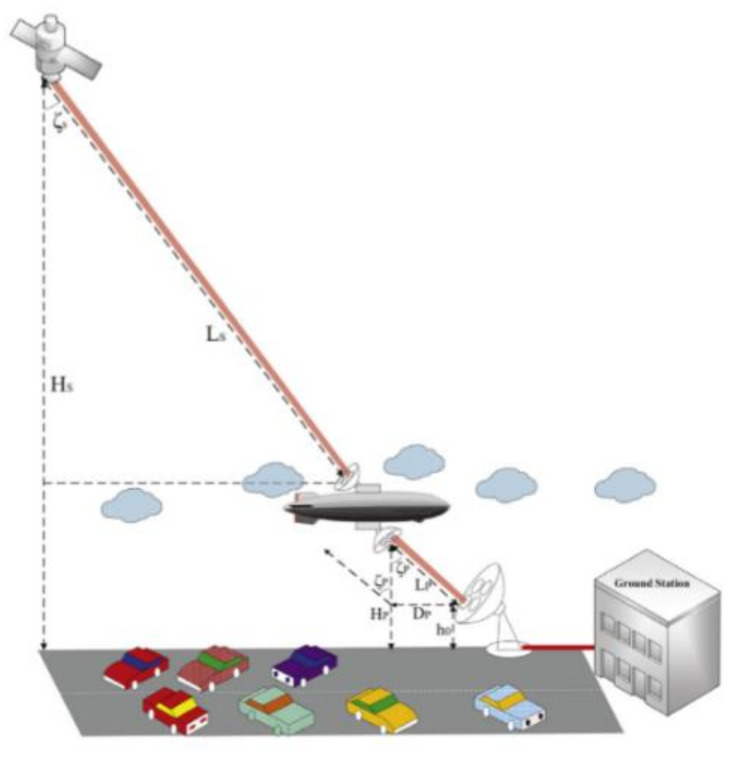
Laser communication scenarios from HAPs [56].

HAPs are quasi-stationary air platforms located in the stratosphere of the atmosphere at an altitude of 17–22 km above the Earth’s surface [52,53,54]. In fact, a high-altitude platform is not a new concept. It usually comes in the form of a hot air balloon and an airship, encased in diatomic hydrogen and helium. Hot air balloons have been used in the past for weather observations and as a simple repeater [53]. However, since balloon flights are not easy to control, they are usually manned or tethered. Furthermore, the HAP-based communication system integrates the advantages of traditional terrestrial communication and satellite communication. On the one hand, it avoids the influence of ground shadow effect and can provide coverage for the area with a diameter of 200–500 km at the same time. On the other hand, it is far superior to satellite communication in terms of payload capacity, deployment cycle, technological difficulty, and cost [55]. HAP can complement and expand ground infrastructure in developed countries and, in some cases, potentially compete with fixed ground and mobile broadband technologies. To be specific, Figure 4 shows the HAP laser communication scenario. Secondly, in terms of specific research progress, Perlot et al. carried out a study on the characteristics of optical channels between HAP and artificial satellites, considering the influence of atmospheric turbulence and cloud cover on the introduction of links [56]. For implementation, Fidler discusses the flexibility of such optical communication links with a wavelength of 1550 nm when the rate requirement reaches 10.7 Gb/s. The results show that the proposed system scheme can improve the link outage probability by more than 10^−12^ and maintain the bit error rate at 10^−9^ with the help of external modulation, return to zero on key control and the lapped optical amplifier with 10 W output power [47]. Besides, HAP, as the relay node between the satellite and the ground terminal, can significantly reduce the processing burden on the satellite, reduce the transmitting power and receiver sensitivity indexes, and bring gains to the overall link budget.

In long-distance transmission, we also need to consider the problem of communication link security; one of the effective ways to solve this problem is to introduce quantum key distribution (QKD) technology into the HAP UAV optical link. QKD enables two partners named Alice and Bob to share a random key that can be used to encrypt/decrypt messages [57]. As early as 2015, the Canadian team reported the first experiment using QKD technology to transmit from launch to satellite, over a distance of 600 km. In the experiment, the challenge of actively correcting beam pointing, photon polarization, and flight time was overcome to generate a 40 bit/s asymptotic security key [58]. This study provides experimental support for secure communication between HAPs and satellites. Subsequently, in 2019, Minh Q. Vu et al. proposed a HAP-assisted relay satellite OWC QKD system to support the safety vehicle network of UAVs and conventional vehicles [59]. In the experiment, the reliability of the system was successfully demonstrated by using the sub-carrier intensity modulation binary phase shift keying (BPSK) and dual threshold/direct detection (DT/DD) receivers, and using the Gaussian beam model to evaluate the influence of geometric expansion on the probability of receiving signals and being eavesdropped by legitimate users. In addition, HAP as a relay node between satellite and ground terminal, can significantly reduce the processing burden on the satellite, reduce transmitting power and receiver sensitivity indicators, and bring gain to the overall link budget.

## 3. Unmanned Aerial Vehicles (UAVs) Optical Wireless Communications

The communication UAVs link is also known as the “air-to-air (A2A)” problem [27,56,60,61,62]. With the development of optical wireless technology in the field of UAVs, more and more researchers realize that a single UAV cannot accomplish tasks independently. The cooperation between UAVs, or even the cooperation among the UAV fleet, can further improve the working quality of UAV technology [28,63]. The development of three dimensional (3D) printing technology also provides strong support for solving the OWC link between UAVs at the hardware level.

### 3.1. Channel Modeling and Parameter Optimization

Establishing an OWC link in the air is an important subject in the research. At the beginning, it was mainly aimed at laser communication between satellites and satellite-to-ground links [64]. Since then, HAP-based optical wireless networks have received great attention. However, there are basic differences between satellite–UAV and UAV-based systems, especially in regards to characteristics of the communication channel [65], which requires special research into the impact of different link parameters on the overall performance.

**Figure 5:**
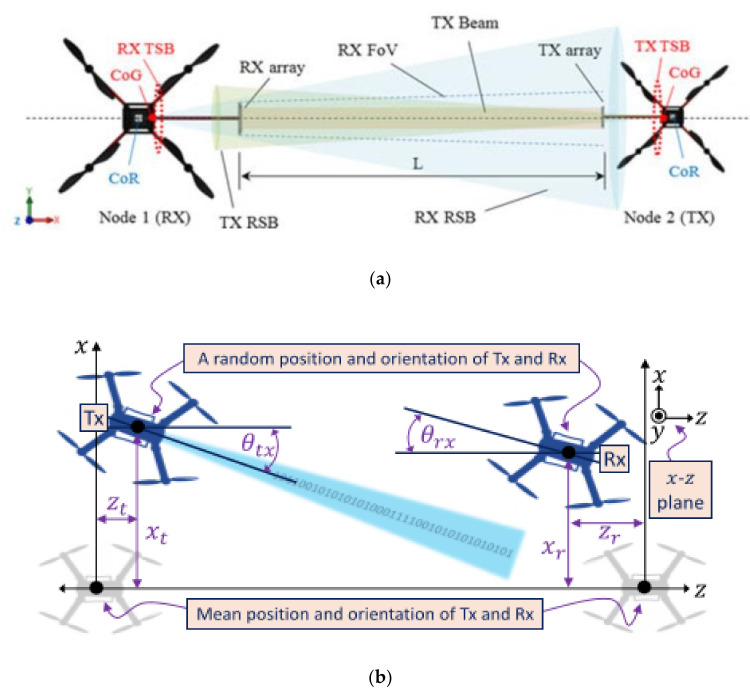
(**a**) A typical A2A free space optical (FSO) link [61] and (**b**) schematic 2D of a UAV–UAV FSO system [27].

For the optical wireless channel modeling between UAVs, as shown in Table 3, there are three main models. They are the atmospheric turbulence model with gamma-gamma logarithmic distribution, the directivity error and atmospheric turbulence composite fading model under the influence of Rayleigh distribution, and the composite attenuation model of energy consumption and atmospheric turbulence in discrete time. In [27], Iranian and British researchers, in cooperation with the weak turbulence environment channel model, use the lognormal turbulence environment channel model to study the UAV optical wireless channel, and then give a closed form expression of the link interruption probability under the weak turbulence condition. In addition, a new closed statistical channel model of gamma-gamma turbulent channel is derived for medium or strong turbulent conditions. Figure 5 is a two dimensional (2D) example of the optical link. The main influencing factors of the above model are the receiving end field angle, spatial position, UAV pointing deviation, et cetera. In this study, similar results are obtained by comparing the obtained results with the traditional time-consuming Monte-Carlo simulation results, thus demonstrating the accuracy of the model. In this model, the researchers put the UAV in hover state. As the UAV is affected by other parameters, its direction and instantaneous position are random, which will deviate from the average position under the ideal model. Under the condition that the link distance of the transceiver terminal is much larger than the absolute value of the instantaneous deviation degree of the UAV in three attitude angles, the researchers considered four effects affecting the signal strength at the receiving end.

They are atmospheric attenuation, atmospheric turbulence, geometric loss, and link interruption caused by signal arrival angle fluctuation. The angle-of-arrival (AOA) of the optical wireless signal refers to the angle between the incident signal at the receiving end and the axial direction of the receiver and the probability distribution function of the AOA can be represented by the Rayleigh distribution. The connection state of the link is represented by “0” and “1”. “0” means interruption and “1” means connection. After comprehensively considering the Rayleigh distribution and link status, the researchers first gave a link interruption state probability distribution function that conformed to the model of the optical communication link between UAVs. Then, added to the optical channel was the atmospheric turbulence and attenuation of influencing factors. They give the complete expression based on optical wireless channel probability distribution function at the end.

In addition, in [62], the researchers also used the gamma-gamma logarithmic distribution atmospheric turbulence model to compare UAVs, the downlink of ground-to-UAV channel, and UAV-to-ground channel models of the uplink. In the case of low turbulence, the output power of the model is far from the Monte-Carlo simulation value, but in the case of moderate or strong turbulence, the output power value is in line with the simulation value. The main reason for this situation is the approximation in [68]. At the same time, the researchers in [27] also optimized the parameters of the optical wireless link between the rotor drones in the hovering state. Through the optimization of the adjustable parameters (including the beam waist and the receiver field of view (FOV) at the transceiver end, the best performance in outage probability was obtained. Relevant numerical results show that the optimal parameters obtained are highly dependent on channel conditions, including: AOA fluctuations, FOV, link distance, turbulence intensity, load jitter, transmission power, and background noise power. Increasing FOV will reduce the output power due to fluctuations in AOA, and a larger FOV will also cause an increase in background noise. Finding suitable FOV parameters is very important for link performance. From the above research, we know that the main influencing factors of a UAV’s communication link are receiver FOV, spatial position, and UAV pointing deviation. Different turbulence intensity leads to different research models. Studying these parameters requires finding a suitable channel model, and the optimized parameters are also highly dependent on channel conditions. This channel model initially expands the ground system into space, and can optimize the optical link parameters of the UAV under different channels or the UAV has randomness.

When people pay more interest in air communication in order to construct aerial optical wireless links between UAVs, especially rotor UAVs, it is necessary to understand the size, load-bearing capacity, stability, and flight status of the UAVs. For the first time in [27], the open loop alignment and stability of a hovering multi-rotor UAV were analyzed. The stability of the UAV communication link is evaluated by the communication distance, wavelength, and platform deviation. Analysis shows that, compared to the impact of translational deviation on the communication link, the rotational deviation has a greater impact on performance, and the system throughput can be stabilized between 16% and 30%. This result can be used as the basic platform characteristics and performance indicators for further research.

### 3.2. Link Performance Enhancement Technology

Link performance is one of the important indicators to evaluate UAV communication performance. In order for UAVs to play better roles in the military and civilian fields, building a high transmission rate, robust, and stable data link is a technical problem we must face. On the one hand, OFDM, as a relatively mature technology and is widely used in indoor OWC systems [70,71,72,73,74]. Based on the development of OFDM technology, researchers proposed to add this technology to the UAV wireless optical system [75,76]. The study thinks about the effects of the log-normal distribution model and the Rayleigh distribution model on the communication system; the closed-form expression of bit error rate (BER) of communication system link is obtained by using the Gauss–Hermite integral method. On this basis, the simulation analyzes the relationship between the beam divergence angle, symbol rate, number of subcarriers, receiving aperture size at the transmitter, and the system BER. It is determined that under the condition of weak turbulence, the laser at the transmitter can make the link error performance reach the optimal beam launch angle, and the influence of the change of turbulence intensity on the optimal beam divergence angle is relatively limited [75]. On the other hand, in the data transmission link, it is necessary to consider the actual rate that the UAV can achieve under the conditions of energy and external interference. Through the research of the Tsinghua research team, we know that when the UAV group increases and the total launch energy is fixed, the data transmission efficiency will increase [75]. One of the reasons for this phenomenon is because there are more drones in the drone swarm to get higher diversity gain. More importantly, when the transmission energy is large and the UAV fleet is large, the coordination of the UAV is more flexible, the transmission power and hovering time can be better allocated, and the data transmission performance can be maximized under the limited interference. In addition, the team also evaluated the impact of the interference power threshold on the data transmission efficiency value. The results show that when the interference power threshold becomes larger, the data transmission efficiency continues to improve. When the interference power threshold is less than 112 dBm, the data transmission efficiency cannot be further improved by increasing the transmission energy. In fact, the reason for this phenomenon is that the transmission power is limited by the interference power. Moreover, when the interference power threshold is large, the data transmission efficiency is saturated, and increasing the transmission energy can significantly improve the data transmission efficiency. This is because when the threshold is increased, the interference power constraint is looser, making the transmission energy the main bottleneck.

**Figure 6:**
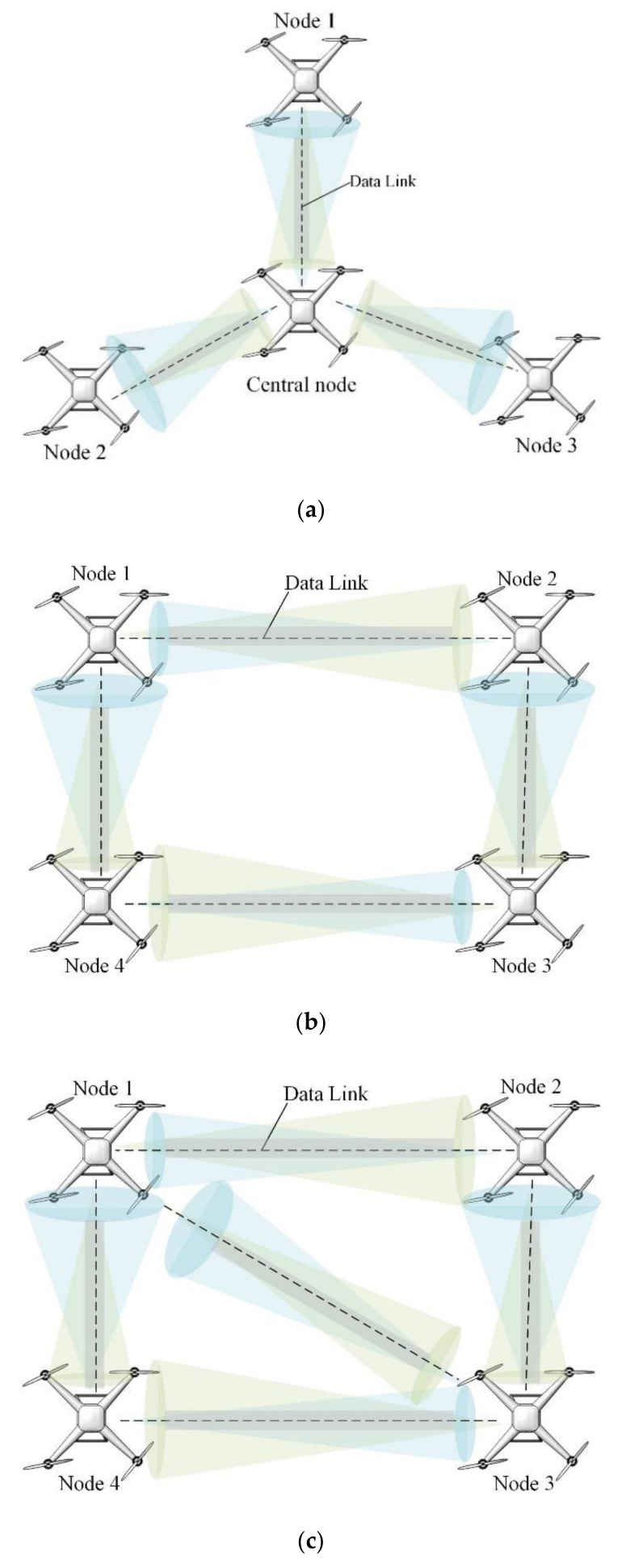
UAV swarm: (**a**) star architecture; (**b**) ring architecture; and (**c**) meshed architecture.

As we know from above, the UAV’s communication links can be enhanced by increasing the number of UAVs. When the number of UAVs reaches a certain level, the link performance can also be further enhanced by considering different types of networking. In terms of the networking architecture of the fixed-wing UAV cluster, as shown in Figure 6, the research team at the Technical University of Graz, Austria, discussed and presented three candidates, Figure 6a is a star architecture, Figure 6b is a ring architecture, and Figure 6c is a meshed architecture. In Figure 6a, the UAV fleet and the OWC configuration form a network of star architectures. The UAV in the middle of the queue acts as an optical multipoint unit (OMU). The remaining users are connected to the OMU through their optical transceiver units. The advantage of this configuration is that the OMU is in the core position and the optical communication link between any two UAVs is shorter. However, when the OMU fails, the entire communication network will be paralyzed. To improve the reliability of the architecture, redundant multi-point units must be installed. In Figure 6b, all UAVs have a two-way link, each with only the front and rear adjacent UAVs, in which case the information is sent in the other direction of the ring network. Therefore, the possibility of failure can be reduced. However, when there is no line of sight path between the transmitter on the UAV and the ground station receiver, multiple intermediate UAVs need to be used as repeaters to establish a link with the ground station. In fact, the ring scheme is a complex technical choice. For high reliability, the best network architecture is a network that combines the advantages of star and ring architectures. In Figure 6c, a mesh network is shown with multiple options for the path of information. The network structure has higher security and reliability in dealing with the failure. However, it must be admitted that multiple connections also increase the complexity of the routing algorithm and increase the difficulty of congestion processing.

Considering the security of communication links, QKD technology can also join in UAV optical links. QKD technology uses the characteristics of quantum mechanics to enable both parties in communication to generate and share a random and secure key to encrypt and decrypt messages. A joint research team from Illinois University and Ohio State University used multiple UAVs to demonstrate the progress of a two-node quantum cryptographic network [77]. In this work, the team focuses on developing a functional optical payload for QKD, which can be used to keep the transmitter and receiver nodes pointed at each other while in flight. Specifically, the UAV rotates automatically to collect data, fixing the position of the detecting beam when it is reflected by another UAV’s angular reflector. At the same time, the QKD protocol was implemented by the introduction of the airborne onboard embedded system (OES) and the alignment of transmitter/receiver nodes was maintained through a fast stabilization system interface with the UAV and optical payload [78].

### 3.3. Module Design and Experiment Test

**Figure 7:**
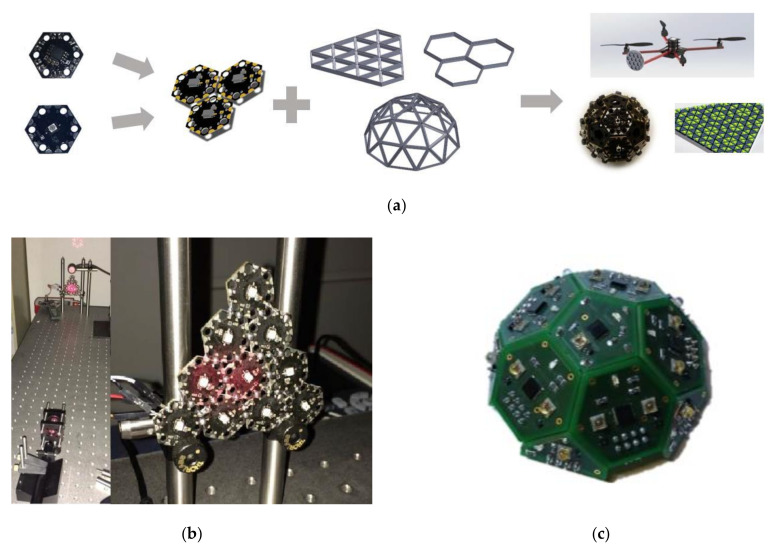
Optical module and experiment scene: (**a**) modular wireless optical elements concept [62]; (**b**) triangular 10-module flat receiver array test [62]; and (**c**) developed optical antenna prototype [79].

It is well known that experimentation is the most direct way to prove theories. In order to further improve the link performance between UAVs, the researchers carried out a modular design on the optical wireless components. Specifically, the research team at the University of Oklahoma used multiple electrical interconnected optical components to form an optical wireless array, mainly planar, hemispherical, and spherical [62]. This flat or curved terminal formed by components has the advantages of low price, light weight, easy assembly, and scalability, and the modules form a continuous surface in a specific polygon, and each module can exist independently as a transceiver. This technology can economically obtain the wide-field optical communication of the last mile broadband connection, and is suitable for optical wireless communication technology applications such as user tracking, spatial diversity reception, multiple inputs and multiple outputs, and detection of beam irregularities caused by interference. As shown in Figure 7a,b, the team also built an example of a triangular planar array test receiver composed of 10 module components. It has been verified that this technology can simultaneously promote the data rate of multiple communication links up to 1 Mb/s.

Fiber bundle technology is a strong candidate technology for OWC, and some research groups have been actively working in this field [67,68,69,70,71,72,78]. The direct function of the optical fiber bundle increases the receiving field of view and realizes space diversity. On this basis, the researchers will install small-sized optical fiber bundles into the optical transmitter and optical receiver of the UAV, respectively. In actual operation, the optical fibers in the optical fiber bundle need to be connected to the multiple lens system on the UAV, to realize the coupling of the optical path, using asynchronous half-duplex or full-duplex serial protocols to communicate.

In addition, researchers from the University of Bremen in Germany also conducted research on optical components. They analyzed the structure of the optical antenna and explored the work related to the modularization of the transceiver [79]. In the experimental prototype that has been completed, the above-mentioned researchers soldered high-power infrared LEDs, photodetectors, and auxiliary circuits to the same hexagonal printed circuit board; a prototype of transceiver antenna is shown in Figure 7c. Furthermore, multiple identical hexagonal optical transceiver circuit boards are combined and spliced into a hemispherical shape. The shape of the hemisphere can provide more radiation angles for the UAV optical wireless communication link. This method improves the reliability of the link to a certain extent.

**Figure 8:**
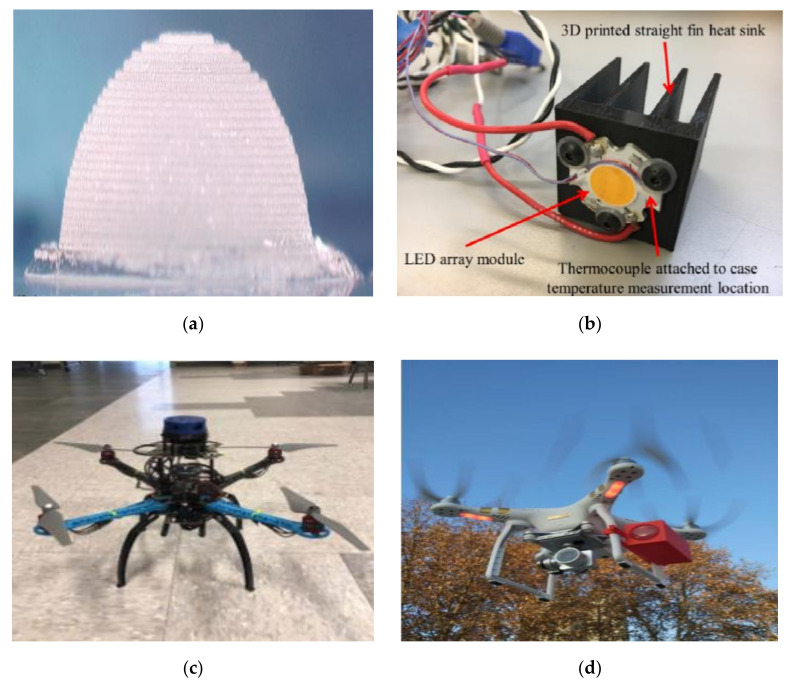
Application of 3D technology: (**a**) 5 mm diameter dome with 2.5 mm dome height on cylinder base of 1.5 mm height [81]; (**b**) 3D printed straight fin heat sink with LED array module mounted [82]; (**c**) UAVs equipped with lidar and 3D printed spiral wings [83]; and (**d**) the pod is tested in the open air [84].

On the other hand, with more products on the market and lower prices, manufacturers are looking for cheaper materials and manufacturing methods. Three-dimensional printing, also known as material manufacturing, is emerging as a low-cost, energy-efficient solution. However, 3D printing is not a new technology. Manufacturers in the automotive, aeronautics and astronautics, and medical industries have long used 3D printing to make parts and systems. Similarly to the 3D printed lens as shown in Figure 8a, the researchers in [80] compared the 3D printed lens with an ordinary LED, and the measurement results of optical output power showed that the lens could increase the output power by 9% when the injected current was 4 mA. In addition, the use of components will lead to the problem of heat loss. It is undeniable that the sensitivity of optical devices changes with the change of temperature. At present, most of the components widely used in heat dissipation systems are metal products. However, these radiators tend to be heavy and expensive on their own and cannot be loaded onto portable platforms such as UAVs. However, with advances in 3D technology, a 3D printed polymer radiator with customized thermal properties can meet the needs of portable mobile platforms.

In [81], researchers studied the thermal conductivity of 3D printed components with different machining directions and percentages of metal filling, and then made straight fin radiator components with filament materials. The LED array is then placed in the radiator to measure the shell temperature of the 3D printed radiator during the use of the LED. Figure 8b shows a 3D printed straight-fin radiator with an LED array module installed and a thermocouple connected to the housing temperature measurement position. The results show that the materials printed with metal filler have higher thermal conductivity than those made with filament without filler. The results also show that the thermal conductivity value depends largely on the printing direction. In terms of the research results of 3D printed optical devices, the research team of Kanguo University in South Korea focused on applying 3D technology to UAVs [82]. The team found that different materials of UAV propellers produce different noises. In the experiment, Figure 8c shows how the team equipped the UAV with reasonably priced optical detection and ranging Lidar sensors and 3D printed propellers of different specifications. Experiments show that the UAV can achieve ideal performance in noise reduction and obstacle detection, and the quality of communication links is also improved. The potential benefits of 3D printed optical components include the ease of creating complex geometric designs and manufacturing speeds. However, how the printing direction and printing resolution affect the transmission and scattering of optical devices has not been studied in detail. UAVs can make it easy for uplink receivers, downlink transmitters, or horizontal link transmitters. The premise is that the UAV can maintain the stability of its own structure, which requires a stable platform that can control the rotation and be installed on the UAV. Considering the payload of the UAV, in [83], the research team of Xi’an Petroleum University printed the photoelectric pod with 3D technology and installed it on the UAV to establish a stable optical link. In Figure 8d, the pod was tested in an open-air scenario. It was initially thought that the weight of the pod would cause the UAV’s fuselage to tilt. However, experiments have shown that the UAV can still fly smoothly after being equipped with the pod. It must be noted that the current angle of the platform’s propagation path still needs to be adjusted manually. The next step is to equip the UAV with an electro-optical pod that can do acquisition, tracking, and pointing (ATP). When ATP is functioning, the UAV platform under 3D technology is a new generation of logistics hardware prototype.

Furthermore, the life of light-source components produced by 3D printing technology is still unclear, which requires further research. However, it can be predicted that with the further development of the research, the products created with 3D printing technology will bring us lower cost and greater utility space.

## 4. Unmanned Aerial Vehicles (UAV) Ground Terminal Optical Wireless Communications

As UAV technology matures, UAVs are becoming more and more common in our lives. UAVs have the characteristics of rapid deployment, flexible structure, and excellent channel conditions, which have attracted much attention. Specially, UAVs can be an effective substitute for temporary or unexpected demand when the ground infrastructure cannot meet the demand for temporary or unexpected traffic, also known as a mismatch between supply and demand. A potential application scenario could be congested highways, where traffic demand for certain sections of the highway increases unexpectedly due to the accumulation of user equipment. In such cases, UAVs can be deployed immediately as a quick response to an emergency.

### 4.1. UAV Connected to a Fixeble-Position Terminal

In the face of major natural disasters, mobile network infrastructure including base stations and power transmission lines will undergo a tremendous test. The communication system is often faced with serious damage, even the emergence of network paralysis and link disruption. Rapid recovery of communication is vital to the rapid transmission of disaster information and the efficient implementation of disaster relief work [85,86]. In existing research, there have been many strategies for restoring communication in the disaster-stricken area. A more common method is to deploy mobile base stations in the disaster area to provide temporary communications for mobile users. Ground vehicular base stations can be deployed near the disaster area, and provide temporary communication by forwarding data between ground mobile base stations located in the disaster area and macro base stations working nearby; in addition, ground vehicle base stations can be moved to different locations upon request [87,88]. However, considering the limitations of practical factors, for example, the road is likely to have been damaged by the disaster, and the vehicle base station cannot reach the destination; in addition, the vehicle base station is generally deployed in the disaster area, close to mobile users, and far away from nearby macro base stations [89,90]. Therefore, the vehicle-mounted base station and the macro base station are often in indirect link conditions, which greatly limit the traffic backhaul capacity of the vehicle-mounted mobile base station.

**Figure 9:**
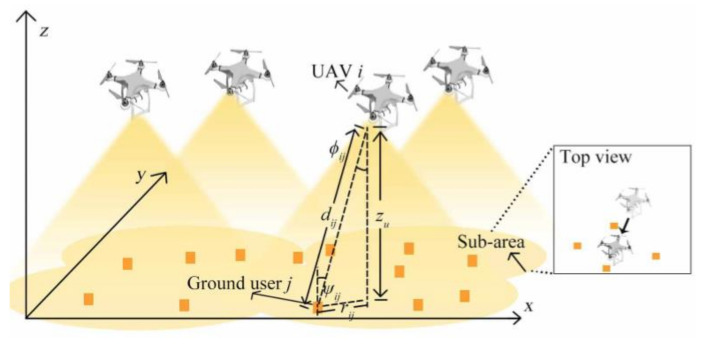
UAV and ground fixed terminal communication scene diagram [7].

In order to cope with the above two types of key issues at the same time, researchers from Virginia Tech and Beijing University of Posts and Telecommunications proposed a deployment problem based on UAV-borne and optical wireless technology. The specific plan is shown in Figure 9. On the one hand, unlike vehicle-mounted base stations, UAVs or UAV base stations can move freely in the air, so they can be efficiently and flexibly deployed in disaster areas without being affected by road conditions on the ground. On the other hand, using optical wireless links to realize point-to-point backhaul between UAVs and neighboring macro base stations can make full use of the high throughput capabilities of optical wireless technology to meet the capacity requirements for backhaul. In addition, on the premise of meeting user rate requirements and maximizing the number of mobile users served, the team proposed a targeted joint optimization algorithm to achieve the three-dimensional spatial location of the UAV base station, mobile user association, and link the dynamic adjustment of the bandwidth allocation with time [7].

Due to optical wireless technology in the outdoor environment, the communication link will face a variety of weather challenges. QKD uses single photon transmission and reception, and the UAV repeatedly sends QKD to receivers on the ground [91]. From their research work [91], we know that the probability of receiving photons decreases sharply as the distance from the transmitter increases, which indicates that the flying height of the UAV should be as low as possible. Secondly, considering that atmospheric loss will affect the reliability of the communication link, within a range of 30 m, the influence of clouds is greater than fog or haze, and when the distance exceeds 30 m, the difference between them is greater. If the UAV altitude can be kept at a relatively low level, changing weather conditions will not cause serious obstacles to the operation of the system [91]. Therefore, UAV-based transmission is a preferred and effective method to transmit QKD to the ground system.

**Figure 10:**
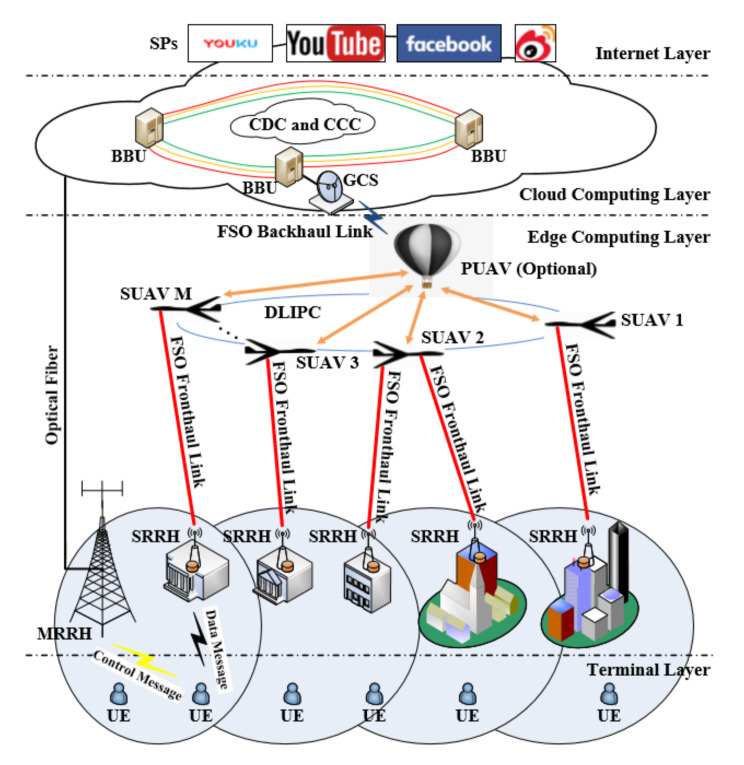
Illustration of the UAV 5G and beyond 5G wireless optical network architecture [8].

In addition, the team from Kazakhstan’s Nazarbayev University systematically assessed the impact of various weather conditions on the vertical optical wireless link between the UAV and the ground station. The results show that compared with rainfall, snowfall has a more significant effect on vertical optical wireless links. Besides, the quantitative results show that with the increase of the specific surface height of the UAV, the effect of geometric loss is further intensified and the link rate performance is further reduced. By comparison, the researchers found that adjusting the beam divergence angle can provide a higher gain than increasing the receiver area in improving the link rate [64]. For the cost and scalability requirements of 5G and beyond 5G wireless networks, researchers from Columbia University proposed a vertical backhaul and forward-transmission frameworks based on optical wireless technology, as shown in Figure 10 [8]. The front and back links are installed on the UAV to achieve fast event response and flexible deployment. The team also studied the architecture’s hardware implementation and networking technology. This framework can be used as a complementary solution to the terrestrial solution, which can significantly improve link Gb-level transmission performance and increase the system’s link margin. However, compared with the cost of the ground network back and forward, the cost of this vertical network is higher. However, there is no doubt that with the maturity of UAV technology and the development of optical wireless technology, vertical networks will make great progress.

### 4.2. UAV Connected to a Variable-Position Terminal

The increase of UAVs altitude will further aggravate the path loss of transmission. The position of UAVs will directly affect the throughput of communication system and the signal-to-noise ratio (SNR) of receiver [92,93]. Therefore, the most important thing is how to find the best distance between the UAV and the user to maximize the link reliability of the vehicle. In [94], it is proved that the position of the UAV on the x, y and z axes directly affects the reliability of A2G link. In [95], the joint position optimization results of multiple UAVs are given to ensure the maximum number of connected users in the system. The authors of [96] studied the impact of UAV height on the average network capacity. It is worth mentioning that in [97], a centralized algorithm is proposed to determine the location of the UAV under the condition of ensuring full coverage of users, to achieve the maximum throughput. The algorithm uses the user’s trajectory to calculate the center of the circle, which gives the motion area (containment area) within which the UAV must be located. The position of the UAV is demarcated by grid points, and the grid points are evaluated by search method to determine the position of the UAV. This UAV positioning algorithm can improve the performance of simulation experiments by 26%. However, the disadvantage of this approach is that the height and the location of the launching end of the UAV are fixed, so it is not possible to evaluate the system throughput in continuous space.

**Figure 11:**
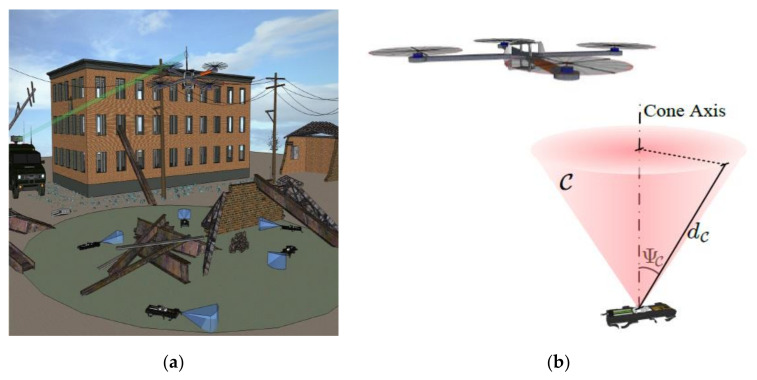
(**a**) A team of micro unmanned aerial and ground vehicles deployed to explore a disaster area; (**b**) connectivity cone C with its parameters [25].

However, the above work is based on RF communication. With the development of optical wireless technology, more and more research teams focus on the optical link between UAVs and ground vehicles. Researchers at the University of New Mexico focused their research on UAVs and ground mobile robots [25]. The UAV carries an optical transmitter, while the ground mobile robot is equipped with an optical receiver. The relative position relationship is quantified by defining the connecting vertebrae of the optical wireless receiver of the ground mobile robot, and its simplified model is shown in Figure 11. 

Because both UAVs and ground mobile robots are in constant motion, it is difficult to maintain the vertebral body model for a long time. The team assumes that the ground robot is moving within a fixed range, and the UAV always knows the state of the ground robot and its linear velocity. Based on these assumptions, a control algorithm is proposed to evaluate the performance of this optical link. The most important part of this algorithm is to determine the position reference point associated with the UAV position on the connecting vertebra. Then according to the relative position vector and relative velocity vector between the position reference point and the UAV, the motion direction of the UAV at the current moment is updated. In brief, so long as the UAV can move inside the vertebral body, it can ensure a reliable connection with the optical link at the ground mobile end. The relative position of the UAV and ground mobile robots has a profound impact on the capacity of optical wireless link between them. The closer the ground mobile robots are to the UAV, the higher the link capacity it can achieve [25]. In this work, the vehicle is connected to the system in a time-division multiplexing way, which greatly alleviates the problem of co-channel interference in the communication system. The position of the UAV is determined by the weighted sum of different vehicle positions. In the process of obtaining the vehicle position weight, it is considered that there is a certain relation between the required descending speed of the vehicle and the vehicle position weight. In the study, the required rate of the user is multiplied by the correction factor, and then put into the Shannon formula to obtain the link distance between the UAV and the vehicle, and take this link distance as the vehicle position weight. Obviously, as the number of vehicles increases, the weighting algorithm becomes more complex. In addition, an increase in the number of vehicles will not result in an increase in the throughput capacity of the above multiple access system. The results show that the capacity gain introduced by the position optimization with the increase of the number of vehicles will decrease successively. When the system maximizes the throughput capacity, up to four vehicles can be connected to the UAV at the same time [42]. There is no denying that the work deserves recognition. However, it must be pointed out that due to the objective existence of atmospheric environment, weather, and obstacles in the traffic environment, the actual communication environment will become bad, and the above work has not considered the influence of objective factors. The performance of optical links under these adverse factors should also be taken into account in future research.

### 4.3. UAV Wireless Light Energy Acquisition

**Figure 12:**
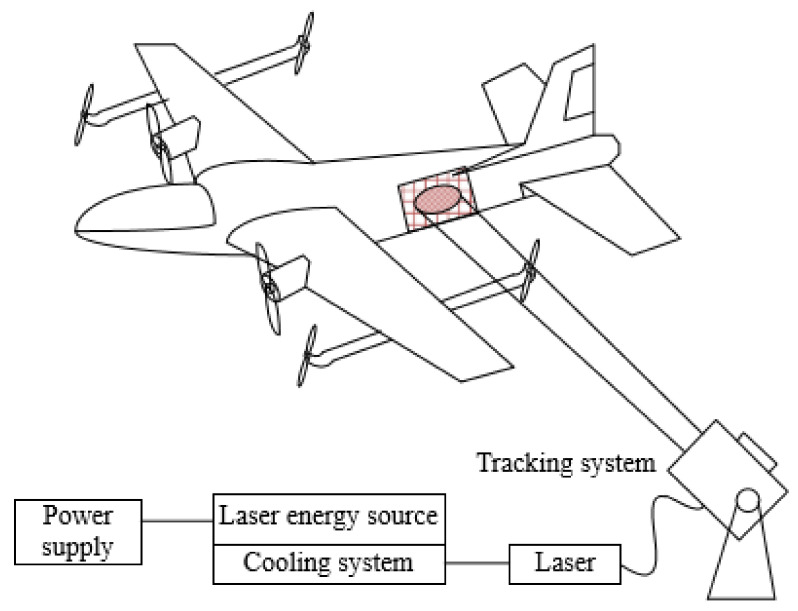
Structure diagram for remote transmission of UAV laser wireless energy [101].

The limited energy carried by the electric UAV can hardly ensure its long-term flight, which seriously affects the performance of its corresponding functions. The advances and applications of laser wireless energy transmission technology have greatly improved the endurance of UAVs. The energy transmission carrier of this technology is laser beam, which receives laser energy through a photovoltaic receiver for photoelectric conversion, thus realizing long-range wireless transmission of energy [98]. Compared to the UAV with a traditional power supply, the laser wireless energy transmission technology improves the endurance of UAV by 24 times [99]. At present, there are relatively few researches on wireless laser power supply technology for UAVs. In 2013, the United States successfully tested the power of a wireless laser charging system using a quad-rotor UAV. Subsequently, the US department of defense plans to develop a new Bat UAV that can fly automatically using laser wireless charging technology [99,100]. As shown in Figure 12, the UAV laser wireless energy transmission system is mainly composed of a ground laser energy transmitter and a laser energy receiver.

It mainly includes: power supply, laser, tracking and pointing system, photoelectric conversion system, and rechargeable battery. Specifically, the laser emitting usually uses a semiconductor laser transmitter. The photoelectric conversion system converts the energy of the laser beam into electrical energy to charge the battery and provide energy for the electric UAV. In the actual application process, on the one hand, we must consider the complex working environment. On the other hand, we need to consider the stability of charging. The most important way to solve these two problems is using ATP algorithms. The ATP algorithm can realize that the UAV maintains high power output during the charging process, improves the charging efficiency, and ensures the stability of the charging. In [101], a laser based on UAV ATP is proposed and designed, consisting of a wireless energy supply system, conducted theoretical analysis, and experimental verification.

**Figure 13:**
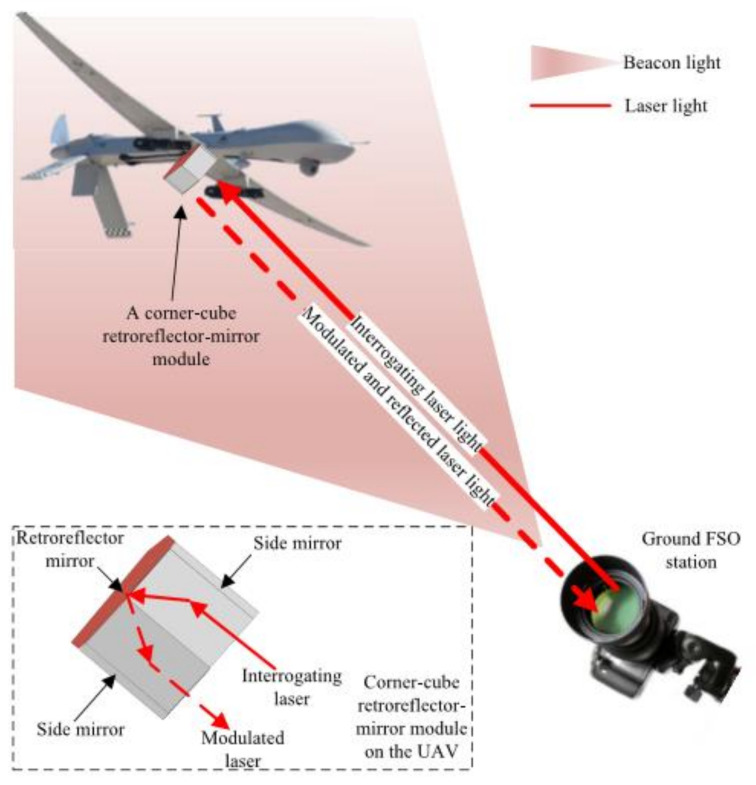
Conceptual illustration of the hybrid ATP mechanism on the ground and a retroreflector mirror mounted onto the UAV to modulate and reflect back an interrogating laser [103].

The main purpose of ATP is not only to obtain the incident optical signal through the transmitter pointing to the receiver, but also to maintain the optical communication link by remotely tracking the position of the optical wireless terminal [102]. Pointing is the process of aiming at the transmitter in the receiver’s FOV. The purpose of the receiver pointing in the direction of beam arrival is the signal acquisition. Tracking is the maintenance of pointing and signal acquisition in the whole process of optical communication between communication terminals. The ATP mechanism is mainly divided into the following types, frame-based, mirror-based, frame-mirror hybrid, adaptive optics, liquid crystal, and RF/FSO hybrid [103]. With different scenarios, different ATPs will choose, for example, a larger pointing range is required for OWC between satellites and drones, whose communication link is the ATP mechanism of framework-based for its first choice [100,101,102,103,104,105,106,107]. However, because of the self-load of the rotating frame, this ATP mechanism is not suitable for the load limitation of small UAVs. However, it has good performance for unmanned vehicles with no weight restrictions [108,109,110,111,112,113]. In the OWC system of railway, the ATP mirror-based mechanism is more commonly used, and the primary problem to be solved is the vibration caused by the train during high-speed operation [114,115,116,117,118]. In [119], the ATP mechanism of micro-electromechanical systems-based and retroreflector-based that was used in UAVs is introduced in detail. The conceptual diagram of this ATP is shown in the Figure 13.

Specifically, the UAV’s rear mirror modulation is coded information that is continuous, and the inquisitive laser from the ground FSO station is reflected-back to the ground. In this ATP mechanism, the reflector can be identified as an angular cube with two side mirrors, global positioning system, for coarse pointing and tracking. A gimbal on the ground continuously tracks the UAV’s trajectory, firing infrared beacons to illuminate the UAV. The ATP mechanism of adaptive optics is studied further based on the astronomical telescope. The liquid crystal has the characteristics of low cost, low power consumption, light weight, and flexible steering components. The ATP mechanism of RF/FSO hybrid can not only solve the problem of LOS link limitation, but also effectively solve the offset problem caused by atmospheric turbulence.

Further, in [119], Bogushevskaya studied different circuit connection methods and proposed a method to improve the laser reception efficiency of UAVs by optimizing the circuit connection mode of photovoltaic receivers on UAVs. Daniel E Becker et al. compared and discussed the conversion efficiency of three photoelectric receivers (flat panel receiver, convergent receiver, and photo-eye), as well as their respective advantages and disadvantages. They concluded that using different photoelectric receivers can improve the laser conversion efficiency of drones under laser irradiation [120]. In the literature [121], Douglas optimized the laser energy distribution and beam convergence based on the rough image of the energy distribution detected by the CCD sensor, and finally achieved the goal of improving transmission efficiency. In literature [17], a laser beam drive system for small and medium-sized electric drones is proposed. The component parts of the system are introduced in detail, and the theoretical framework of the system is established. All the above documents are the theoretical derivation of the UAV luminous wireless power supply system and related optimization research of photovoltaic receivers. However, there are few studies on the charging efficiency and stability of the laser energy supply onboard UAVs. Therefore, it is necessary to conduct further research into related technologies.

### 4.4. Experimental Prototypes and Testing Activities

At present, researchers in related fields have begun to carry out experimental testing of optical wireless links between UAVs and ground terminals. Table 4 summarizes the experimental progress of optical wireless links between some UAVs and ground terminals (including indoor optical wireless communications (IOWC) and outdoor optical wireless communications). Several units, including China Electronics Technology Group, have carried out preliminary experimental work. In the experiment, the UAV is fixed at a position 6.7 km away, and the image is transmitted to the ground receiving end through the optical wireless communication link. The highest transmission rate during the experiment can reach 1.25 Gb/s [122]. In addition, researchers at the Korea Institute of Electronics and Telecommunications have designed optical terminals for shared paths between drones and ground terminals. The design scheme uses integrated optical components to simultaneously support the transmission of optical beams and beacon beams. The initial experimental results show that the terminal can support a 1.25 Gb/s full-duplex error-free link, and the static transmission distance can reach 50 m [40]. Based on the above work, it is worth noting that although one end of the above-mentioned experimental transceiver is a UAV platform, in the specific optical wireless communication process, the transceiver is always limited to a fixed position. In other words, the link performance of the UAV in flight remains to be further studied.

In addition, in recent years, due to the improvement of image sensor technology, researchers have also tried to use the camera as a receiving element of the optical wireless link. In [126], the communication performance of the ground sensor based on OWC technology to set up the optical transmission link between the UAV under the background radiation such as sunlight was studied. In this study, the transmission power at the ground end is fixed, the communication performance is analyzed by calculating the optical power at the receiving end, and the OWC link can achieve a bit rate in Gb/s by measuring the data. On the one hand, the team in Okayama, Japan, is trying to install a camera on a helicopter UAV that uses a camera to capture quick response (QR) code information on an LED display. The two-dimensional code information involved mainly includes the QR code and augmented reality marker (AR) code [56]. By extracting the spatial location information contained in the two-dimensional code information, the UAVs can be assisted to operate according to the required location. The team believes the above options are part of an automated flight system. On the other hand, the proposed system in [127] includes a ground transmitter and a UAV, which increases capacity through an orbit-angle-momentum multiplexing multi-orbital angular momentum beam, and a collaboration with the UAV OWC link to discuss the damage of the UAV’s communication channel under stationary, hovering, and motion conditions. The team demonstrated and described the performance of the system on multiplexed OWC links from an experimental perspective.

**Figure 14:**
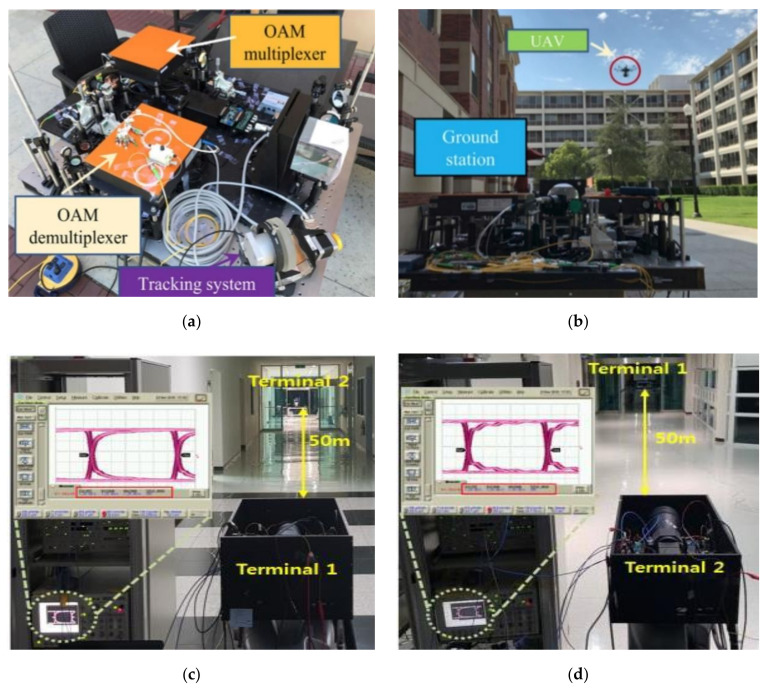
Photos of experimental equipment. (**a**) Ground station; (**b**) communication link between the ground transmitter and the ground receiver via the UAV [127]; (**c**) BER measurement of the WOC link for UAVs (Terminal 1) and GCSs (Terminal 2) from Terminal 1 to Terminal 2; and (**d**) from Terminal 2 to Terminal 1 [128].

As can be seen from Figure 14a, the orbital angular momentum (OAM) transmitter, the OAM receiver, and the beam tracking system are integrated into a ground station, which is placed on the lawn side.

As shown in Figure 14b, the UAV carries a reflector on the other side of the lawn until it is 50 m from the ground station. During the test, the drone takes off, climbs, moves to a specific position and hovers, and finally lands on the ground; Figure 14c shows the drone hovering over the scene during the experiment. By reusing 2 OAM beams, each beam carries 40 Gb/s QPSK signals, enabling a total capacity of up to 80 Gb/s on up to 100 round-trip links. The results of the experiment have shown that the required mode of power fluctuates by 2.1 dB when the drone hovers in the air, while the power of crosstalk in other modes is less than the desired mode of −19 dB. In addition, channel crosstalk decreases as the OAM mode interval increases. Based on the above research, researchers at the University of Southern California in the United States attempted to introduce orbital angular momentum technology and multi-input multi-output equalization technology into the design of optical wireless links between drones and ground stations. The experimental results are as shown in Figure 14c,d, and the results show that the two-channel 40 Gb/s A2G transmission performance can be achieved by means of the modulation of the aircraft, and the round-trip distance of the reverse modulation link can reach 100 m. The comparative experiment shows that multi-input and multi-output (MIMO) equalization can significantly reduce the effect of the turbulence effect on optical wireless link degradation, and reduce the error bit rate to 3.8 × 10^−3^ [29]. It is foreseeable that the wireless data throughput capacity of drones can be further enhanced as more enabling technologies are introduced into the field of optical wireless links in the air space.

## 5. Unmanned Aerial Vehicles (UAV)-Sea Surface Optical Wireless Communications

**Figure 15:**
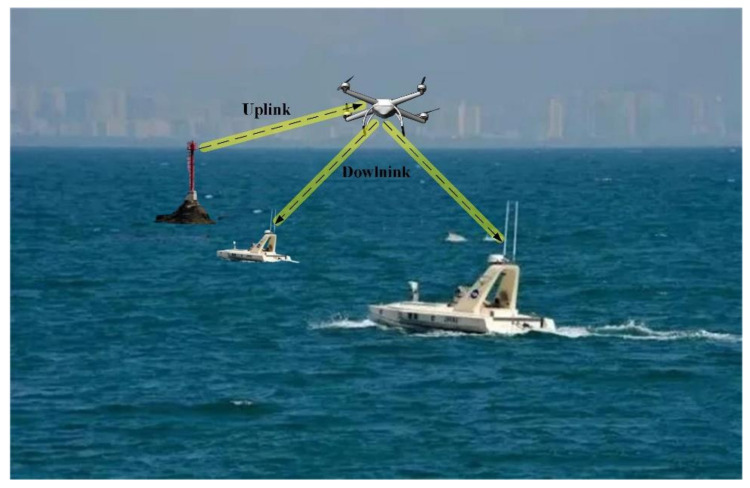
UAV–sea surface OWC scenario, arrows indicate the direction of data transmission.

Compared with the development of satellite and terrestrial 5G communications, there is a big gap in broadband coverage at sea. A feasible scheme is to realize the potential benefits of maritime communication, and the specific network diagram is shown in Figure 15. In this network, the UAV is used to provide real-time coverage for mobile ships.

### 5.1. Trajectory Optimization and Performance Analysis

UAVs have huge potential for coverage enhancement. UAVs can play different roles in different scenarios. The major difference between UAV communication and traditional communication is the controllable maneuverability of UAV. When the UAV is hovering in the coverage area, its advantages mainly depend on the height of the UAV [129]. When using a mobile UAV, its trajectory should be the most important research object [130,131,132]. However, the current research is approaching simplification, and the main problems are the power supply of the UAV, simple circular trajectory, et cetera. [129,130,131,132,133,134]. The trajectory of the UAV should be adjusted according to the position of the moving user, to maximize the speed of each condition [135,136].

In [137], Li optimized the trajectory and launch power of the UAV to minimize the ship’s achievable speed. In contrast to his previous work, he considered a typical composite channel model involving both large-scale and small-scale fading to simulate the actual propagation environment. However, because the UAV cannot get the dynamic small-scale fading accurately before take-off, only the large-scale channel state information (CSI) is optimized to analyze the communication performance in this model; the constraints of kinematics and communication are considered in the optimization. The simulation results show that the proposed UAV coverage superiority can greatly optimize the communication performance of the sea surface network. Xian University of Posts and Telecommunications team derived the mathematical expression of the average BER of the VLC system by studying the VLC system of marine ships and land lighthouses. They also analyzed the link performance of the VLC system, wave fluctuation, transmission distance, receiver aperture size, atmospheric turbulence intensity, and optical wavelength [138]. Similarly, we can think of the sea-to-ground fixed-end optical channel as a communication channel between hovering UAVs and sea-surface terminals. The results show that the performance of the lighthouse–ship VLC system is affected by the wave, and the variation of the average BER is as random and complex as the wave. The average BER of VLC system increases with the increase of transmission distance and atmospheric turbulence intensity, and decreases with the decrease of receiver aperture and wavelength. The results can be used as a reference for the design and performance evaluation of ship optical communication system.

### 5.2. Hybrid Link Analysis

When UAVs build optical wireless links with sea facilities, they must perform real-time altitude adjustments to meet the requirements of line of sight (LOS) communication. When calculating the positional deviation between the UAV and the sea surface facility, the scintillation characteristics of the ocean–atmospheric channel will cause errors in the altitude adjustment algorithms in the existing research. If there is no change in the intensity of the received light signal caused by atmospheric flicker, when the intensity of the received light signal of the drone becomes weak, it means that the distance between the drone and the surface buoy becomes longer, and it is necessary to adjust the altitude or make subtle changes by position adjustment. However, the presence of atmospheric flicker usually weakens the intensity of the optical signal. If the UAV also adjusts the previous altitude or position at this time, it will destroy the normal LOS communication. Therefore, the scintillation effect caused by atmospheric turbulence brings uncertainty to the original altitude adjustment algorithm. On the one hand, an adjustment factor can be introduced into the algorithm to compensate, first eliminating the influence of flicker, and then adjusting the position or altitude, to ensure the reliability of the received signal and the smooth communication link.

On the other hand, a hybrid communication link can be used to compensate. In practical applications, the resistance of the UAV’s optical wireless link to weather conditions is lower than that of the traditional wireless link. The UAV needs to improve the beam pointing, acquisition, and tracking during the movement to ensure the line of sight data link. For the above two types of problems, researchers from the Institute of Optoelectronics of the Polish Military University of Technology proposed to combine optical wireless links with traditional RF links [139]. RF/FSO communication links can enhance data transmission throughput, real-time receiving capabilities, and reduce network energy consumption [140,141,142,143]. In the research center of the team, they divided the RF/FSO communication mode into direct mode and retroreflective mode. In direct mode, two FSO transceivers are used to provide high bandwidth. However, the size, weight, and power of this configuration may not be suitable for simple platforms such as small unmanned aerial vehicles and unmanned sensors. For these applications, retro-reflector mode will be a better solution [139]. This hybrid link model has not been proposed for the first time. Hybrid system constructions were designed to provide better availability, bandwidth, and security, or no-complex configuration [144,145].

A significant problem with the above model is that the RF band is limited, and the resources of the optical network have not been fully developed. Millimeter-wave (mm Wave) and fiber optic systems can provide higher data rate networks [18,146]. In response to this advantage, the research team of the National Institute of Information and Communication Technology of Japan and the School of Science and Engineering of Waseda University in Tokyo provided an ultra-fast, energy-saving, and low-latency communication solution [146]. This solution combines optical fiber wireless transmission, free space wireless transmission, and millimeter wave wireless transmission system to successfully transmit 40 Gb/s mm Wave signals in the 100 GHz frequency band. Specifically, in this method, the network center generates light mm Wave signals, and transmits the signals to the relay node through the optical fiber link, and then transmits it to the free space by the wireless optical terminal. During transmission, OWC signals can be received and seamlessly converted into millimeter-wave radio signals. Mm Wave signals are directly transmitted to end users without other conversion and processing. This transmission mode reduces system complexity, power loss, and delay. At the same time, the team mentioned in the article that in future work, efficient beam control and coupling technology will be developed to study how to efficiently convert OWC signals into mm Wave signals. Although this research has made some progress, this work is aimed at indoor transmission systems. In the outdoor environment, changes in the atmospheric environment also need to be considered. The joint research team of the Technical University of Prague in the Czech Republic and the Institute of Multimedia Telecommunications of the Polytechnic University of Valencia in Spain has further researched the above system. The team analyzed the link performance under the FSO channel at 500 m through simulation experiments, and obtained a transmission rate of Gb/s [147]. From these two studies, we know that in future mobile networks this technology can bring better application prospects, which also provides a new direction for the research of drones. What we can foresee is that applying this work to UAV systems will bring greater benefits.

## 6. Comparative Analysis and Trend Outlook

In modern society, communication runs through all aspects of our lives. It brings great convenience to our daily lives and plays a huge role in social development. However, in the event of natural disasters or terrorist attacks and other human events, communication base stations and other infrastructure will often be destroyed, which is a large obstacle to saving people’s lives and property when emergency communications arise. How to establish an efficient emergency communication system is an important problem to reduce the damage and maintain social stability. In recent years, the research and application of using UAVs to build emergency communication systems have appeared one after another, which shows that the UAV technology provides an efficient solution for the construction of emergency communication systems. At present, there are not many researches on UAV communication networks in large-scale emergency communication scenarios, and there are areas for improvement.

Specifically, from the previous table, we can know that the current research direction is mainly for A2G communication links, and the research on A2A communication links and A2S communication links is limited. The most popular optical wavelength is 1550 nm, and the channel type is mostly free space optical channel. In terms of transmission rate, fixed-wing UAVs can usually achieve Gb/s transmission performance due to their advantages in payload. In terms of meter-level short-distance A2G links, considering factors such as flight flexibility, cost, size, and power consumption, researchers are more inclined to choose multi-rotor UAVs as the basic airborne platform. At the same time, due to the significant shortening of the link distance, the optical signal emitted by the transmitter no longer needs to experience traditional long-distance fading effects such as atmospheric turbulence, atmospheric scattering, and beam drift; therefore, reducing security risks. To increase the link coverage, the researchers replaced the source of this type of link with infrared or visible light-emitting diodes. In the numerical simulation of this type of link, the corresponding channel type is usually replaced with Lambertian optical wireless communication.

There are some obvious gaps in the existing research. Some key problems and potential solutions that restrict airborne optical wireless links need to be discussed further. At the present stage, the assumptions in the partial link numerical simulation are too ideal. The researchers have not fully explored some of the main parameters that affect the UAV’s communication links. When planning a UAV’s trajectory, most consider all UAVs flying at the same fixed altitude, and a follow up study could look at when the UAV’s altitude is inconsistent. In other words, when the UAV can fly anywhere in 3D space, how does the UAV get the position information or channel state information of the ground receiving terminal, how to measure, what kind of uplink is used for feedback, and how real-time and accuracy of measurement and feedback all need further discussion. In addition, the existing UAVs emergency communication network combines the communication coverage and the connectivity between UAVs. Although there are three main networking modes: star, ring, and meshed architecture, the communication network is not necessarily the best, because some links of the UAV emergency communication network may cause the problem of heavy traffic load, which could easily block the efficient transmission of data. Therefore, if the load distribution of each link after the network is considered and the communication coverage and traffic load are balanced, the UAV emergency communication network will be more efficient.

In our life, because of the complexity of the geographical environment and the distribution of people, there have been studies that considered the uneven distribution from the consistent distribution of users on the ground, but this is only preliminary work. More complex scenarios, such as more diverse user profiles, high mountains and other obstacles, and critical locations such as rescue stations and emergency command centers, could be considered for follow up studies; all these factors have a significant impact on the optimal deployment of UAVs. At present, the research of large-scale UAV emergency communication networks is focused on the static deployment of UAV communication networks, but ground users can move at any time, and their distribution will change accordingly. There are many researches on the dynamics of UAV networks, which can be used for reference to the framework and algorithm of UAV mobile control, and deeply study the position change of UAVs in emergency communication networks with the change of users on the ground.

Regarding link capacity, the existing research is limited to a large extent to the single-wavelength link configuration, and lack of full mining of rich spectral resources. In traditional optical fiber communication and terrestrial FSO communication systems, the wavelength division multiplexing (WDM) can increase link capacity exponentially by transmitting optical signals of different optical carriers in the same optical channel. This kind of multi-wavelength link configuration can be introduced into airborne optical wireless systems to achieve significant capacity gain while taking into account the payload capability of UAVs. On the other hand, we can try to introduce multi-input and multi-output technology to improve the reliability and spectrum efficiency of the link. At present, the existing research work usually adopts the single-input-single-output transceiver configuration; that is, only one light source transmitter and one photoelectric detector. Once the transceiver links are affected by turbulence, beam drift, occlusion, and other adverse factors, the transmission performance will deteriorate rapidly or even link interruption. By introducing a plurality of light source emitters at the transmitter and a plurality of photodetectors at the receiver, a plurality of spatial links can objectively be built between the transmitter and the receiver. Even if the performance of one link is degraded, the overall performance is still relatively reliable due to the presence of other links, thus largely avoiding link interruption. At the same time, by introducing space–time coding, singular value decomposition, and other spatial multiplexing algorithms, we can transmit different content data streams on different transmit-receive links, thus improving the spectral efficiency of MIMO airborne wireless optical links.

Besides, the main problem of UAV optical wireless communication is to realize the ATP mechanism between the transmitter and receiver. The deviation line displacement of the optical signal at the receiving end is proportional to the angular displacement of the signal transmitting end, and the proportional coefficient is the link communication distance. Therefore, the greater the deviation of the signal transmitting end, the lower the probability that the receiving end can receive information correctly, putting forward very high requirements for the ATP mechanism. One feasible way is to use non-Lambertian light sources instead of traditional Lambertian light sources. In fact, many LED manufacturers have been able to reshape the light beams emitted by LEDs with the help of technologies such as packaging and adding secondary optical lenses to obtain non-Lambertian or even customized light beam effects. In the design process of the airborne wireless optical link, by introducing non-Lambertian light sources, it is possible to construct diversified link options in the optical beam dimension and improve the capability of ATP. In the next study, the rationalization of the solutions to the above problems will greatly affect the practical process of the UAV field.

## 7. Conclusions

In recent years, with the application of 3D printing technology, energy supply technology, and path planning technology to UAVs, the performance indicators and application scope of UAVs have been greatly improved. No matter the industrial field or special application fields, UAVs play an important role. The OWC technology has the advantages of rich spectrum resources, strong directionality, high transmission capacity, and resistance to RF electronic interference. The introduction of OWC technology into the field of UAVs can greatly improve the data transmission and carrying capacity of various UAVs. Based on this technology, academia and industry pay more attention to the relationship of UAV–satellite terminals, UAVs, UAV–ground terminals, and UAV–sea surface terminals. They proposed and discussed the channel model and link characteristics between the UAV and the communication terminal. With the continuous deepening of research, the application potential of OWC technology in the field of UAVs will be developed.

## Figures and Tables

**Table 1:** The types of UAV optical wireless modulation.

Author	Electric Field Modulation	Optical Field Modulation	Wavelength	Distance	Ref
X. J. Huang et al.	QAM and ACO-OFDM	MZM	-	200 km	[6]
Lajos Hanzo et al.	PPM and OFDM	IM	940 nm	-	[14]
P. L. Chen et al.	OOK	MZM	1550 nm	2.16 km	[21]
Marzieh Najafi et al.	OOK	IM	1550 nm	500 m	[22]
Dissanayake S D et al.	OOK	IM	254 nm	1000 m	[23]
J. X. An et al.	OOK	IM	1543 nm	6.76 km	[24]
Patricio J. et al.	OOK	IM	White light	100 m	[25]
Figueiredo M et al.	QAM and DCO-OFDM	IM	White light	-	[26]
Mohammad T D et al.	OOK	IM	White light	250 m	[27]
Ramdhan N et al.	QPSK and CDMA	MZM	1548 nm	2ߝ4 km	[28]
L. Li et al.	QPSK	IM	1550 nm	1 km	[29]
Amantayeva A et al.	OOK	IM	-	20 m	[37]
D. Wu et al.	QAM and OFDM	IM	1550 nm	2 km	[38]

**Table 2:** Research on A2S experiment test.

UAV Type	Link Type	Distance	Channel	Wavelength	Data Rate	Work Type	Ref
Fixed-wing	A2S	~36,000 km	LOS	1550 nm	1.8 Gb/s	Simulation	[46]
HAP	A2S	35,000 km	LOS	1550 nm	10.7 Gb/s	Simulation	[47]
HAP	A2S	-	LOS	-	10 Gb/s	Simulation	[48]
HAP	A2S	20 km	LOS	1550 nm	10 Gb/s	Simulation	[49]
HAP	A2S	517 km	LOS	1550 nm	2.5 Gb/s	Simulation	[50]

**Table 3:** Air-to-air (A2A) experimental channel model.

Types	Ref
Gamma-gamma logarithmic distribution atmospheric turbulence model	[27,56,63,64,66,67]
Directivity error and atmospheric turbulence compound fading model subject to Rayleigh distribution	[60,68]
Discrete-time energy consumption and atmospheric turbulence compound fading model	[69]

**Table 4:** Comparison of performance and parameters for UAV airborne optical wireless links.

UAV Type	Link Type	Distance	Channel	Wavelength	Data Rate (Gb/s)	Work Type	Ref
Fixed-wing	A2G	300 m	LOS	750 nm	-	Simulation	[8]
Fixed-wing	A2G	1–20 km	LOS and NLOS	1550 nm	Gb/s	Simulation	[9]
-	A2G	500 m	LOS	1550 nm	Gb/s	Simulation	[22]
Multi-rotor	A2G	1000 m	LOS	254 nm	5 kb/s	Simulation	[23]
Multi-rotor	A2G	20 km	LOS	1550 nm	1.13	Simulation	[25]
Multi-rotor	A2G	100 m	LOS	1550 nm	40	Experiment	[29]
Multi-rotor	A2G	~10 m	LOS and NLOS	White light	10 Mb/s	Simulation	[40]
Multi-rotor	A2G	~20 m	LOS	White light	~10 Mb/s	Simulation	[42]
Multi-rotor	A2G	2 km	LOS	600 nm	Gb/s	Simulation	[123]
-	A2G	0.2–6 m	LOS	1550 nm	-	Simulation	[124]
Multi-rotor	A2G	100 m	LOS	-	1 Gb/s	Simulation	[125]

## Abbreviations

The following abbreviations are used in this manuscript:

2D	2 Dimensional
3D	3 Dimensional
5G	Fifth generation
6G	Sixth generation
A2A	Air-to-air
A2G	Air-to-ground
A2S	Air-to-satellite
AR	Augmented reality
ACO-OFDM	Asymmetrically clipped optical orthogonal frequency division multiplexing
ATP	Acquisition, tracking, and pointing
AOA	Angle-of-arrival
BER	Bit error rate
BPSK	Binary phase shift keying
CCD	Charge coupled device
CDMA	Code division multiple access
CSI	Channel state information
DC	Direct current
DT/DD	Dual threshold/direct detection
DC-OFDM	Direct current orthogonal frequency division multiplexing
FSO	Free space optical
FOV	Field of view
HAP	High-altitude platform
IM	Intensity modulation
IOWC	Indoor optical wireless communication
LAP	Low-altitude platform
LiFi	Light fidelity
LED	Light-emitting diode
LOS	Line of sight
MIMO	Multi-input and multi-output
MZM	Mach–Zehnder modulation
mm Wave	Millimeter-wave
OAM	Orbital angular momentum
OES	Onboard embedded system
OFDM	Orthogonal frequency division multiplexing
OMU	Optical multipoint unit
OWC	Optical wireless communication
OCC	Optical camera communication
OOK	On/off keying
PV	Photovoltaic
PPM	Pulse position modulation
QKD	Quantum key distribution
QAM-4	Pulse amplitude modulation-4
QAM	Quadrature amplitude modulation
QPSK	Quadrature phase shift keying
QR	Quick response
RF	Radio frequency
RSU	Roadside units
SNR	Sight-of-noise ratio
UAV	Unmanned aerial vehicle
VLC	Visible light communication
WDM	Wavelength division multiplexing
